# Supplementary material for: Influence of organic, synthetic and biofertilizers on the diversity of cassava rhizosphere microbiome in Northeastern Thailand
Source: PeerJ. 2025 Oct 3;13:e20085. doi: 10.7717/peerj.20085 (PMC12499567; doi:10.7717/peerj.20085)
Supplement: Supplemental Information 1 [file peerj-13-20085-s001.docx]

**Table S1** Summary of the number of base sequences at each step of amplicon sequence variant (ASV) construction using the DADA2 program for 16S rRNA gene sequences from each soil sample from around cassava tubers at 2, 5, and 10 MAP grown at the Nampong site.

| Time of soil sample Collection | Sample name | Input | Filtered | Percentage of input passed filter | Denoised | Non-chimeric | Percentage of input non-chimeric |
| --- | --- | --- | --- | --- | --- | --- | --- |
| 2 MAP | T1M2_NP047 | 193156 | 161063 | 83.38 | 130767 | 84533 | 43.76 |
|  | T1M2_NP134 | 189819 | 159441 | 84 | 135590 | 87845 | 46.28 |
|  | T1M2_NP307 | 193172 | 163207 | 84.49 | 125998 | 95406 | 49.39 |
|  | T2M2_NP247 | 189059 | 159153 | 84.18 | 121008 | 91494 | 48.39 |
|  | T2M2_NP394 | 191769 | 161819 | 84.38 | 125929 | 93081 | 48.54 |
|  | T2M2_NP669 | 191888 | 160370 | 83.57 | 132937 | 103239 | 53.8 |
|  | T3M2_NP274 | 201381 | 163737 | 81.31 | 131633 | 103911 | 51.6 |
|  | T3M2_NP494 | 191939 | 165326 | 86.13 | 109586 | 96842 | 50.45 |
|  | T3M2_NP752 | 189655 | 154311 | 81.36 | 116701 | 86945 | 45.84 |
|  | T4M2_NP027 | 190887 | 161651 | 84.68 | 131226 | 93427 | 48.94 |
|  | T4M2_NP594 | 199567 | 167286 | 83.82 | 118138 | 103487 | 51.86 |
|  | T4M2_NP772 | 201076 | 164229 | 81.68 | 129664 | 98250 | 48.86 |
|  | T5M2_NP112 | 185838 | 152896 | 82.27 | 105324 | 90659 | 48.78 |
|  | T5M2_NP407 | 189952 | 157020 | 82.66 | 104348 | 90342 | 47.56 |
|  | T5M2_NP629 | 191103 | 162744 | 85.16 | 115697 | 91181 | 47.71 |
|  | T6M2_NP067 | 191438 | 163064 | 85.18 | 138226 | 103215 | 53.92 |
|  | T6M2_NP149 | 193305 | 164078 | 84.88 | 124119 | 100673 | 52.08 |
|  | T6M2_NP612 | 190269 | 158174 | 83.13 | 126993 | 111619 | 58.66 |
|  | T7M2_NP509 | 189802 | 161340 | 85 | 131530 | 92090 | 48.52 |
|  | T7M2_NP554 | 194048 | 168569 | 86.87 | 148171 | 94772 | 48.84 |
|  | T7M2_NP787 | 189421 | 158735 | 83.8 | 120668 | 101493 | 53.58 |
|  | T8M2_NP012 | 188737 | 155986 | 82.65 | 124150 | 88735 | 47.02 |
|  | T8M2_NP232 | 188784 | 154413 | 81.79 | 130585 | 113786 | 60.27 |
|  | T8M2_NP449 | 192099 | 158624 | 82.57 | 134574 | 109258 | 56.88 |
| 5 MAP | T1M5-NP054 | 122480 | 98906 | 80.75 | 92141 | 89748 | 73.28 |
|  | T1M5-NP347 | 144544 | 123694 | 85.58 | 117005 | 114374 | 79.13 |
|  | T1M5-NP732 | 160763 | 138076 | 85.89 | 131902 | 128497 | 79.93 |
|  | T2M5-NP252 | 150036 | 123807 | 82.52 | 116606 | 113262 | 75.49 |
|  | T2M5-NP389 | 148808 | 134082 | 90.1 | 126274 | 125023 | 84.02 |
|  | T2M5-NP694 | 156572 | 131407 | 83.93 | 125290 | 122068 | 77.96 |
|  | T3M5-NP169 | 174322 | 149874 | 85.98 | 142351 | 139709 | 80.14 |
|  | T3M5-NP489 | 153940 | 134330 | 87.26 | 127477 | 125041 | 81.23 |
|  | T3M5-NP747 | 159844 | 130136 | 81.41 | 122339 | 120131 | 75.16 |
|  | T4M5-NP034 | 157103 | 134810 | 85.81 | 128535 | 125893 | 80.13 |
|  | T4M5-NP289 | 154621 | 134900 | 87.25 | 127308 | 124352 | 80.42 |
|  | T4M5-NP774 | 155763 | 115112 | 73.9 | 109805 | 107776 | 69.19 |
|  | T5M5-NP107 | 152001 | 136061 | 89.51 | 128229 | 125292 | 82.43 |
|  | T5M5-NP327 | 152212 | 134342 | 88.26 | 127050 | 124743 | 81.95 |
|  | T5M5-NP412 | 140750 | 113635 | 80.74 | 105189 | 102502 | 72.83 |
|  | T6M5-NP069 | 149207 | 133076 | 89.19 | 125558 | 124448 | 83.41 |
|  | T6M5-NP094 | 203622 | 167600 | 82.31 | 160504 | 156981 | 77.09 |
|  | T6M5-NP607 | 178149 | 152974 | 85.87 | 144655 | 142158 | 79.8 |
|  | T7M5-NP434 | 158787 | 139360 | 87.77 | 130717 | 127718 | 80.43 |
|  | T7M5-NP547 | 160373 | 142475 | 88.84 | 136488 | 133511 | 83.25 |
|  | T7M5-NP792 | 170478 | 152370 | 89.38 | 145211 | 142541 | 83.61 |
|  | T8M5-NP009 | 164512 | 140235 | 85.24 | 133730 | 132149 | 80.33 |
|  | T8M5-NP454 | 175110 | 154221 | 88.07 | 146926 | 144967 | 82.79 |
|  | T8M5-NP572 | 181847 | 161655 | 88.9 | 154372 | 152029 | 83.6 |
| 10 MAP | T1M10_NP052 | 161294 | 145842 | 90.42 | 140753 | 136293 | 84.5 |
|  | T1M10_NP132 | 161454 | 146531 | 90.76 | 141586 | 138368 | 85.7 |
|  | T1M10_NP312 | 171085 | 155734 | 91.03 | 150695 | 146481 | 85.62 |
|  | T2M10_NP249 | 159384 | 144188 | 90.47 | 138656 | 134235 | 84.22 |
|  | T2M10_NP672 | 175589 | 160442 | 91.37 | 155450 | 150913 | 85.95 |
|  | T2M10_NP692 | 175501 | 157032 | 89.48 | 151775 | 147572 | 84.09 |
|  | T3M10_NP172 | 177134 | 162127 | 91.53 | 156914 | 154152 | 87.03 |
|  | T3M10_NP212 | 227245 | 173901 | 76.53 | 166519 | 161325 | 70.99 |
|  | T3M10_NP272 | 176321 | 161923 | 91.83 | 156872 | 152852 | 86.69 |
|  | T4M10_NP032 | 164418 | 151712 | 92.27 | 146331 | 141983 | 86.35 |
|  | T4M10_NP287 | 155792 | 141936 | 91.11 | 136323 | 132812 | 85.25 |
|  | T4M10_NP649 | 157384 | 144218 | 91.63 | 137995 | 134522 | 85.47 |
|  | T5M10_NP332 | 164129 | 149786 | 91.26 | 144074 | 140541 | 85.63 |
|  | T5M10_NP372 | 157358 | 141896 | 90.17 | 137074 | 133185 | 84.64 |
|  | T5M10_NP632 | 178282 | 162837 | 91.34 | 156965 | 154330 | 86.57 |
|  | T6M10_NP072 | 165814 | 149389 | 90.09 | 143282 | 139998 | 84.43 |
|  | T6M10_NP152 | 165346 | 151703 | 91.75 | 145554 | 141970 | 85.86 |
|  | T6M10_NP614 | 192267 | 156249 | 81.27 | 148785 | 145021 | 75.43 |
|  | T7M10_NP432 | 174034 | 156945 | 90.18 | 151216 | 146796 | 84.35 |
|  | T7M10_NP514 | 83344 | 75413 | 90.48 | 70921 | 69497 | 83.39 |
|  | T7M10_NP532 | 156930 | 143074 | 91.17 | 137163 | 133075 | 84.8 |
|  | T8M10_NP227 | 156033 | 141172 | 90.48 | 134771 | 131356 | 84.18 |
|  | T8M10_NP472 | 161719 | 148284 | 91.69 | 142508 | 139527 | 86.28 |
|  | T8M10_NP574 | 179340 | 149615 | 83.43 | 142579 | 140257 | 78.21 |
