# Supplementary material for: Influence of organic, synthetic and biofertilizers on the diversity of cassava rhizosphere microbiome in Northeastern Thailand
Source: PeerJ. 2025 Oct 3;13:e20085. doi: 10.7717/peerj.20085 (PMC12499567; doi:10.7717/peerj.20085)
Supplement: Supplemental Information 2 [file peerj-13-20085-s002.docx]

**Table S2** Summary of the number of base sequences at each step of amplicon sequence variant (ASV) construction using the DADA2 program for 16S rRNA gene sequences from each soil sample from around cassava tubers at 2, 5, and 10 MAP grown at the Seungsang site.

| Time of soil sample collection | Sample name | Input | Filtered | Percentage of input passed filter | Denoised | Non-chimeric | Percentage of input non-chimeric |
| --- | --- | --- | --- | --- | --- | --- | --- |
| 2 MAP | T1M2_SS052 | 184920 | 139534 | 75.46 | 90381 | 82067 | 44.38 |
|  | T1M2_SS331 | 183890 | 139762 | 76 | 84295 | 78003 | 42.42 |
|  | T1M2_SS507 | 188576 | 148923 | 78.97 | 101801 | 84833 | 44.99 |
|  | T2M2_SS128 | 184740 | 150671 | 81.56 | 102484 | 89674 | 48.54 |
|  | T2M2_SS489 | 198159 | 153177 | 77.3 | 99162 | 87598 | 44.21 |
|  | T2M2_SS697 | 184341 | 142499 | 77.3 | 100120 | 85995 | 46.65 |
|  | T3M2_SS268 | 186463 | 151458 | 81.23 | 97764 | 86928 | 46.62 |
|  | T3M2_SS433 | 187678 | 154698 | 82.43 | 103367 | 93281 | 49.7 |
|  | T3M2_SS749 | 183674 | 139723 | 76.07 | 85611 | 78295 | 42.63 |
|  | T4M2_SS027 | 198718 | 163438 | 82.25 | 114925 | 100871 | 50.76 |
|  | T4M2_SS187 | 191074 | 153572 | 80.37 | 99261 | 87745 | 45.92 |
|  | T4M2_SS673 | 203922 | 160943 | 78.92 | 109895 | 96902 | 47.52 |
|  | T5M2_SS114 | 192464 | 159142 | 82.69 | 102628 | 89316 | 46.41 |
|  | T5M2_SS356 | 185398 | 144441 | 77.91 | 96378 | 85689 | 46.22 |
|  | T5M2_SS532 | 193708 | 157734 | 81.43 | 102314 | 91886 | 47.44 |
|  | T6M2_SS008 | 187317 | 150584 | 80.39 | 108710 | 88868 | 47.44 |
|  | T6M2_SS624 | 187177 | 154123 | 82.34 | 108393 | 96777 | 51.7 |
|  | T6M2_SS785 | 191355 | 154429 | 80.7 | 99282 | 89412 | 46.73 |
|  | T7M2_SS297 | 187835 | 148617 | 79.12 | 102808 | 86473 | 46.04 |
|  | T7M2_SS373 | 198140 | 161841 | 81.68 | 101508 | 92408 | 46.64 |
|  | T7M2_SS588 | 195585 | 154865 | 79.18 | 96579 | 87152 | 44.56 |
|  | T8M2_SS210 | 187653 | 146161 | 77.89 | 89629 | 79636 | 42.44 |
|  | T8M2_SS384 | 186604 | 141797 | 75.99 | 93751 | 85016 | 45.56 |
|  | T8M2_SS558 | 204297 | 167276 | 81.88 | 108041 | 96264 | 47.12 |
| 5 MAP | T1M5-SS054 | 158647 | 128684 | 81.11 | 122033 | 120051 | 75.67 |
|  | T1M5-SS510 | 154209 | 130898 | 84.88 | 123998 | 121842 | 79.01 |
|  | T1M5-SS708 | 169089 | 148110 | 87.59 | 141510 | 140072 | 82.84 |
|  | T2M5-SS135 | 155605 | 140967 | 90.59 | 132987 | 131356 | 84.42 |
|  | T2M5-SS222 | 150533 | 130994 | 87.02 | 123912 | 121556 | 80.75 |
|  | T2M5-SS457 | 113549 | 98883 | 87.08 | 93348 | 91829 | 80.87 |
|  | T3M5-SS267 | 160184 | 142023 | 88.66 | 134052 | 131889 | 82.34 |
|  | T3M5-SS432 | 157442 | 138310 | 87.85 | 130538 | 128672 | 81.73 |
|  | T3M5-SS562 | 142254 | 121253 | 85.24 | 114589 | 113035 | 79.46 |
|  | T4M5-SS193 | 166752 | 145458 | 87.23 | 137967 | 135484 | 81.25 |
|  | T4M5-SS469 | 147743 | 130622 | 88.41 | 125566 | 123589 | 83.65 |
|  | T4M5-SS671 | 157066 | 129487 | 82.44 | 123339 | 121509 | 77.36 |
|  | T5M5-SS104 | 154238 | 138510 | 89.8 | 131839 | 130315 | 84.49 |
|  | T5M5-SS310 | 148564 | 131509 | 88.52 | 124715 | 122997 | 82.79 |
|  | T5M5-SS767 | 132576 | 115570 | 87.17 | 110458 | 108514 | 81.85 |
|  | T6M5-SS003 | 147942 | 130213 | 88.02 | 123376 | 121960 | 82.44 |
|  | T6M5-SS176 | 173229 | 152724 | 88.16 | 146439 | 143529 | 82.86 |
|  | T6M5-SS420 | 155476 | 136567 | 87.84 | 130083 | 128014 | 82.34 |
|  | T7M5-SS377 | 139090 | 116213 | 83.55 | 109057 | 107576 | 77.34 |
|  | T7M5-SS583 | 144649 | 127798 | 88.35 | 121503 | 120120 | 83.04 |
|  | T7M5-SS646 | 140628 | 112358 | 79.9 | 105354 | 103947 | 73.92 |
|  | T8M5-SS062 | 122555 | 106550 | 86.94 | 99439 | 97787 | 79.79 |
|  | T8M5-SS209 | 159770 | 130567 | 81.72 | 123202 | 120862 | 75.65 |
|  | T8M5-SS545 | 129498 | 107822 | 83.26 | 101467 | 99769 | 77.04 |
| 10 MAP | T1M10_SS053 | 169873 | 154263 | 90.81 | 149170 | 145551 | 85.68 |
|  | T1M10_SS336 | 140550 | 125167 | 89.06 | 119428 | 117572 | 83.65 |
|  | T1M10_SS711 | 151923 | 130575 | 85.95 | 125237 | 122905 | 80.9 |
|  | T2M10_SS121 | 157680 | 140946 | 89.39 | 135369 | 132402 | 83.97 |
|  | T2M10_SS459 | 168234 | 147779 | 87.84 | 142362 | 138873 | 82.55 |
|  | T2M10_SS482 | 147426 | 127049 | 86.18 | 121763 | 118841 | 80.61 |
|  | T3M10_SS082 | 151343 | 135736 | 89.69 | 129552 | 127139 | 84.01 |
|  | T3M10_SS579 | 129440 | 58383 | 45.1 | 54882 | 54038 | 41.75 |
|  | T3M10_SS759 | 153425 | 134837 | 87.88 | 128568 | 125486 | 81.79 |
|  | T4M10_SS022 | 164307 | 144828 | 88.14 | 139465 | 136570 | 83.12 |
|  | T4M10_SS616 | 157758 | 129991 | 82.4 | 124434 | 122549 | 77.68 |
|  | T4M10_SS668 | 152470 | 130379 | 85.51 | 124621 | 122157 | 80.12 |
|  | T5M10_SS109 | 150089 | 134409 | 89.55 | 128708 | 125929 | 83.9 |
|  | T5M10_SS538 | 160878 | 134105 | 83.36 | 128308 | 125457 | 77.98 |
|  | T5M10_SS772 | 167780 | 150568 | 89.74 | 143951 | 139863 | 83.36 |
|  | T6M10_SS010 | 165050 | 148527 | 89.99 | 143077 | 140826 | 85.32 |
|  | T6M10_SS166 | 159094 | 143542 | 90.22 | 137723 | 134360 | 84.45 |
|  | T6M10_SS792 | 158739 | 130553 | 82.24 | 124391 | 121139 | 76.31 |
|  | T7M10_SS144 | 112153 | 99667 | 88.87 | 95217 | 93448 | 83.32 |
|  | T7M10_SS284 | 173050 | 152473 | 88.11 | 147511 | 144468 | 83.48 |
|  | T7M10_SS641 | 158038 | 139434 | 88.23 | 133550 | 130901 | 82.83 |
|  | T8M10_SS063 | 152223 | 138126 | 90.74 | 131825 | 129782 | 85.26 |
|  | T8M10_SS557 | 182232 | 139550 | 76.58 | 133158 | 131897 | 72.38 |
|  | T8M10_SS739 | 154168 | 137756 | 89.35 | 131658 | 129194 | 83.8 |
