# Supplementary material for: Influence of organic, synthetic and biofertilizers on the diversity of cassava rhizosphere microbiome in Northeastern Thailand
Source: PeerJ. 2025 Oct 3;13:e20085. doi: 10.7717/peerj.20085 (PMC12499567; doi:10.7717/peerj.20085)
Supplement: Supplemental Information 3 [file peerj-13-20085-s003.docx]

**Table S3** Observed features data of soil microorganisms around the tubers at 2, 5, and 10 MAP from Nampong (M2_NP, M5_NP, and M10_NP, respectively) and Seungsang (M2_SS, M5_SS, and M10_SS, respectively)

| Time and sites | Average mean | Number | Std. Deviation |
| --- | --- | --- | --- |
| M2_NP | 1005.42 | 24 | 293.547 |
| M5_NP | 2729.21 | 24 | 363.501 |
| M10_NP | 2593.04 | 24 | 311.507 |
| M2_SS | 1445.50 | 24 | 179.380 |
| M5_SS | 2557.46 | 24 | 348.499 |
| M10_SS | 2274.71 | 24 | 270.957 |
| Total | 2100.89 | 144 | 712.531 |
