# Supplementary material for: Influence of organic, synthetic and biofertilizers on the diversity of cassava rhizosphere microbiome in Northeastern Thailand
Source: PeerJ. 2025 Oct 3;13:e20085. doi: 10.7717/peerj.20085 (PMC12499567; doi:10.7717/peerj.20085)
Supplement: Supplemental Information 4 [file peerj-13-20085-s004.docx]

**Table S4** Comparison of alpha diversity among treatments (T1-T8) using the Kruskal-Wallis (pairwise) test for soil around cassava tubers grown at the Nampong (NP) and Seungsang (SS) sites revealed significant differences between treatments.

| Time and sites | Group 1 | Group 2 | H value | p-value | q-value |
| --- | --- | --- | --- | --- | --- |
| 5 MAP at NP | T1 | T3 | 3.8571 | 0.0495 | 0.0841 |
|  | T1 | T5 | 3.8571 | 0.0495 | 0.0841 |
|  | T1 | T6 | 3.8571 | 0.0495 | 0.0841 |
|  | T1 | T8 | 3.8571 | 0.0495 | 0.0841 |
|  | T4 | T6 | 3.8571 | 0.0495 | 0.0841 |
|  | T5 | T6 | 3.8571 | 0.0495 | 0.0841 |
| 10 MAP at NP | T1 | T3 | 3.8571 | 0.0495 | 0.0841 |
|  | T2 | T3 | 3.8571 | 0.0495 | 0.0841 |
| 2 MAP at SS | T1 | T4 | 3.8571 | 0.0495 | 0.0841 |
|  | T1 | T5 | 3.8571 | 0.0495 | 0.0841 |
|  | T1 | T8 | 3.8571 | 0.0495 | 0.0841 |
|  | T2 | T4 | 3.8571 | 0.0495 | 0.0841 |
|  | T4 | T5 | 3.8571 | 0.0495 | 0.0841 |
| 5 MAP at SS | T3 | T4 | 3.8571 | 0.0495 | 0.0841 |
|  | T3 | T7 | 3.8571 | 0.0495 | 0.0841 |
|  | T3 | T8 | 3.8571 | 0.0495 | 0.0841 |
|  | T6 | T7 | 3.8571 | 0.0495 | 0.0841 |
|  | T6 | T8 | 3.8571 | 0.0495 | 0.0841 |
| 10 MAP at SS | T1 | T4 | 3.8571 | 0.0495 | 0.0841 |
|  | T4 | T8 | 3.8571 | 0.0495 | 0.0841 |
