# Supplementary material for: Influence of organic, synthetic and biofertilizers on the diversity of cassava rhizosphere microbiome in Northeastern Thailand
Source: PeerJ. 2025 Oct 3;13:e20085. doi: 10.7717/peerj.20085 (PMC12499567; doi:10.7717/peerj.20085)
Supplement: Supplemental Information 5 [file peerj-13-20085-s005.docx]

**Table S5** Bacterial genera identified from the soil surrounding cassava tubers at the Nampong and Seungsang sites, 2, 5, and 10 months after planting (MAP).

| Identified bacterial genera | | |
| --- | --- | --- |
| 2 MAP | 5 MAP | 10 MAP |
| 1_Unclassified Acidobacteriales  2_uncultured Acidobacteria bacterium  3_Uncultured bacterium in Acidobacteriales  4_Bryobacter  5_Candidatus Solibacter  6_Uncultured bacterium in Blastocatellia (Subgroup 4)  7_RB41  8_Uncultured bacterium in Acidobacteria  9_Uncultured bacterium in Acidimicrobiia  10_Mycobacterium  11_Nocardia  12_Acidothermus  13_Glycomyces  14_Unclassified Intrasporangiaceae  15_Microbacterium  16_Pseudarthrobacter  17_Isoptericola  18_Unclassified Micromonosporaceae  19_Micromonospora  20_Nocardioides  21_Microlunatus  22_Saccharomonospora  23_Streptomyces  24_Actinomadura  25_Uncultured bacterium in Actinobacteria  26_Rubrobacter  27_Gaiella  28_Uncultured bacterium in Gaiellales  29_Uncultured bacterium in Solirubrobacterales  30_Conexibacter  31_Chitinophaga  32_Terrimonas  33_Ohtaekwangia  34_Uncultured bacterium in Microscillaceae  35_Dyadobacter  36_Flavobacterium  37_Chryseobacterium  38_Unclassified Sphingobacteriales  39_Sphingobacterium  40_Uncultured bacterium in Anaerolineae  41_Herpetosiphon  42_Uncultured bacterium in Roseiflexaceae  43_Uncultured bacterium in Chloroflexi  44_Tumebacillus  45_Unclassified Bacillaceae  46_Bacillus  47_Fictibacillus  48_Gracilibacillus  49_Oceanobacillus  50_Virgibacillus  51_Ammoniphilus  52_Brevibacillus  53_Paenibacillus  54_Lysinibacillus  55_Staphylococcus  56_Kroppenstedtia  57_Thermoactinomyces  58_Clostridium sensu stricto 1  59_Romboutsia  60_Uncultured bacterium in Gemmatimonadaceae  61_Nitrospira  62_Dongia  63_Uncultured bacterium in Elsterales  64_Microvirga  65_Devosia  66_Allorhizobium-Neorhizobium-Pararhizobium-Rhizobium  67_Ensifer  68_Mesorhizobium  69_Ochrobactrum  70_Shinella  71_Bradyrhizobium  72_Uncultured bacterium in Xanthobacteraceae  73_Sphingobium  74_Sphingomonas  75_Bdellovibrio  76_Unclassified bacteriap25  77_Unclassified Burkholderiaceae  78_Azohydromonas  79_Burkholderia-Caballeronia-Paraburkholderia  80_Comamonas  81_Cupriavidus  82_Massilia  83_Ralstonia  84_Roseateles  85_Unclassified Enterobacteriaceae  86_Enterobacter  87_Klebsiella  88_Kosakonia  89_Acidibacter  90_Acinetobacter  91_Pseudomonas  92_Steroidobacter  93_Stenotrophomonas  94_Candidatus Udaeobacter  95_Uncultured bacterium in WPS-2 | 1_Uncultured Acidobacteria bacterium  2_Uncultured bacterium in Acidobacteriales  3_Bryobacter  4_Candidatus Solibacter  5_RB41  6_Uncultured bacterium in Acidobacteria  7_Uncultured bacterium in Acidimicrobiia  8_Uncultured Ilumatobacteraceae  9_Uncultured bacterium in Microtrichales  10_Catenulispora  11_Mycobacterium  12_Nocardia  13_Acidothermus  14_Jatrophihabitans  15_Geodermatophilus  16_Microbacterium  17_Micromonospora  18_Uncultured Micromonosporaceae  19_Nocardioides  20_Microlunatus  21_Crossiella  22_Pseudonocardia  23_Kitasatospora  24_Streptomyces  25_Actinomadura  26_Uncultured bacterium in Actinobacteria  27_Rubrobacter  28_Gaiella  29_Uncultured bacterium in Gaiellales  30_Uncultured bacterium in Solirubrobacterales  31_Conexibacter  32_Solirubrobacter  33_Dysgonomonas  34_Uncultured Microscillaceae  35_Flavobacterium  36_Uncultured Roseiflexaceae  37_Uncultured bacterium Thermomicrobiales  38_Uncultured bacterium in Chloroflexi  39_Uncultured bacterium in Ktedonobacteria  40_Uncultured bacterium in Ktedonobacterales  41_1921-2  42_FCPS473  43_Uncultured Ktedonobacteraceae  44_Uncultured bacterium in Chloroflexi  45_Microcoleus SAG 1449-1a  46_Tumebacillus  47_Bacillus  48_Listeria  49_Ammoniphilus  50_Paenibacillus  51_Sporolactobacillus  52_Lactobacillus  53_Weissella  54_Clostridium sensu stricto 1  55_Clostridium sensu stricto 12  56_Gemmatimonas  57_Uncultured Gemmatimonadaceae  58_Nitrospira  59_Azospirillum  60_SWB02  61_Dongia  62_Uncultured bacterium in Elsterales  63_Reyranella  64_Microvirga  65_Pedomicrobium  66_Allorhizobium-Neorhizobium-Pararhizobium-Rhizobium  67_Bradyrhizobium  68_Uncultured Xanthobacteraceae  69_Sphingomonas  70_Anaeromyxobacter  71_Uncultured bacterium in Myxococcales  72_Haliangium  73_Uncultured Sandaracinaceae  74_Uncultured bacterium in Deltaproteobacteria  75_Burkholderia-Caballeronia-Paraburkholderia  76_Comamonas  77_Cupriavidus  78_Massilia  79_Ralstonia  80_MND1  81_Uncultured bacterium in Gammaproteobacteria  82_Cellvibrio  83_Enterobacter  84_Klebsiella  85_Acidibacter  86_Pseudomonas  87_Arenimonas  88_Pseudoxanthomonas  89_Uncultured bacterium in Rokubacteriales  90_Candidatus_Udaeobacter  91_Verrucomicrobium  92_Uncultured bacterium in WPS-2 | 1_Uncultured Acidobacteria bacterium  2_Uncultured bacterium in Acidobacteriales  3_Bryobacter  4_Candidatus Solibacter  5_RB41  6_Uncultured bacterium in Acidobacteria  7_Uncultured bacterium in Acidimicrobiia  8_Uncultured Ilumatobacteraceae  9_Uncultured bacterium in Microtrichales  10_Mycobacterium  11_Nocardia  12_Acidothermus  13_Jatrophihabitans  14_Blastococcus  15_Geodermatophilus  16_Agromyces  17_Microbacterium  18_Pseudarthrobacter  19_Cellulosimicrobium  20_Isoptericola  21_Catellatospora  22_Dactylosporangium  23_Micromonospora  24_Kribbella  25_Nocardioides  26_Microlunatus  27_Crossiella  28_Pseudonocardia  29_Saccharopolyspora  30_Streptomyces  31_Nonomuraea  32_Actinomadura  33_Uncultured bacterium in Actinobacteria  34_Rubrobacter  35_Gaiella  36_Uncultured bacterium in Gaiellales  37_Uncultured bacterium in Solirubrobacterales  38_Conexibacter  39_Solirubrobacter  40_Uncultured Roseiflexaceae  41_Uncultured bacterium in Thermomicrobiales  42_Uncultured bacterium in Chloroflexi  43_Uncultured bacterium in Ktedonobacterales  44_FCPS473  45_Uncultured bacterium in Chloroflexi  46_Tumebacillus  47_Bacillus  48_Fictibacillus  49_Ammoniphilus  50_Paenibacillus  51_Lysinibacillus  52_Uncultured Gemmatimonadaceae  53_Uncultured bacterium in Longimicrobiaceae  54_Dongia  55_Uncultured bacterium in Elsterales  56_Reyranella  57_Microvirga  58_Allorhizobium-Neorhizobium-Pararhizobium-Rhizobium  59_Mesorhizobium  60_Ochrobactrum  61_Nordella  62_Bradyrhizobium  63_uncultured Xanthobacteraceae  64_Sphingomonas  65_Burkholderia-Caballeronia-Paraburkholderia  66_Massilia |
