# Supplementary figures and images for: Influence of organic, synthetic and biofertilizers on the diversity of cassava rhizosphere microbiome in Northeastern Thailand
Source: PeerJ. 2025 Oct 3;13:e20085. doi: 10.7717/peerj.20085 (PMC12499567; doi:10.7717/peerj.20085)

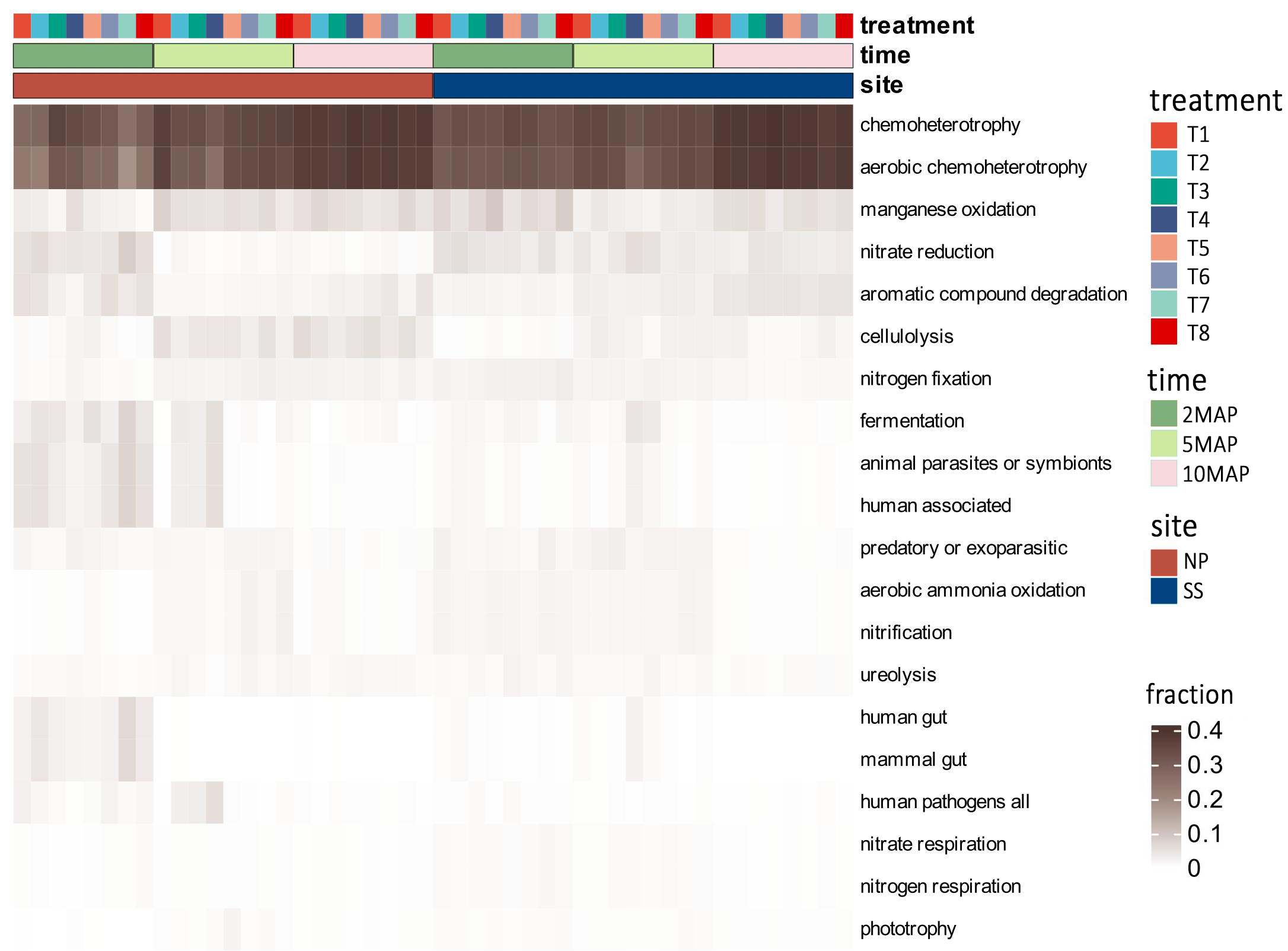

Supplement: Supplemental Information 7 — Heatmap illustrating the fractional representation of functional pathways predicted by FAPROTAX in microbial communities from different treatments (T1–T8), sampling times (2, 5, and 10 MAP), and sites (NP and SS). The intensity of the shading corresponds to the fraction of each functional category. [file peerj-13-20085-s007.jpg]
